# DBFOX-Ph/metal complexes: Evaluation as catalysts for enantioselective fluorination of 3-(2-arylacetyl)-2-thiazolidinones

**DOI:** 10.3762/bjoc.4.16

**Published:** 2008-05-20

**Authors:** Takehisa Ishimaru, Norio Shibata, Dhande Sudhakar Reddy, Takao Horikawa, Shuichi Nakamura, Takeshi Toru

**Affiliations:** 1 Department of Frontier Materials, Graduate School of Engineering, Nagoya Institute of Technology, Gokiso, Showa-ku, Nagoya 466-8555, Japan

**Keywords:** fluorination, enantioselective, nickel, Lewis acid, catalyst

## Abstract

We examined the catalytic enantioselective fluorination of 3-(2-arylacetyl)-2-thiazolidinones **1** with *N*-fluorobenzenesulfonimide (NFSI) by DBFOX-Ph/metal complexes under a variety of conditions. After optimization of the metal salts, solvents and additives, we found that the fluoro-2-thiazolidinones **2** were obtained in good to high yields with moderate to good enantioselectivities (up to 78% ee) when the reaction was carried out in the presence of DBFOX-Ph (11 mol%), Ni(ClO_4_)_2_·6H_2_O (10 mol%) and 2,6-lutidine (0 or 1.0 equiv) in CH_2_Cl_2_.

## Background

**Figure 1:**
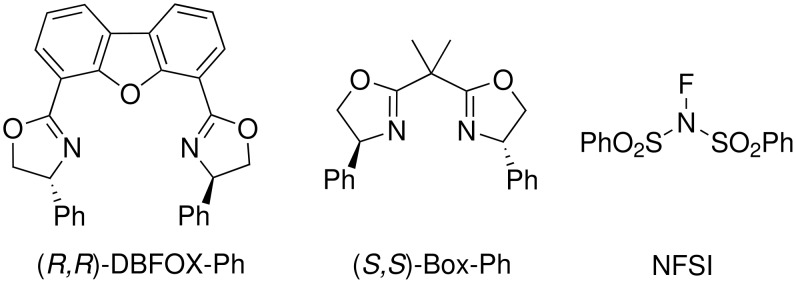
Structures of DBFOX-Ph, Box-Ph and NFSI.

Enantioselective electrophilic fluorination represents an important and straightforward strategy for C-F bond formation at a carbon stereocenter, providing easy access to chiral fluoro-organic compounds [1–2]. Due to the significance of chiral fluoro-organic compounds, such as fluorinated quinolones [3–4] and liquid crystals [5], in pharmaceutical and material sciences considerable effort has been dedicated to this issue for decades [6–17]. As a consequence, a variety of procedures have been developed to increase the yields and enantioselectivities of electrophilic fluorination reactions. Stoichiometric approaches based on cinchona alkaloid/Selectfluor® combinations [18–32], chiral ligand/metal-catalyzed [33–57] or organocatalytic [58–64] procedures for enantioselective fluorination are major advances in recent years. The discovery that chiral ligands/metals can catalyze electrophilic fluorination with conventional fluorinating reagents has had a large impact on synthetic organic chemistry, because of the availability of commonly used classes of ligands for asymmetric catalysis, such as, TADDOLs [37,39,41,47], BINAPs [38,40,43–44,46,49,51,53,55–57] and bis(oxazoline) [33–34,36,42,45]. Of particular importance are BINAP ligands. Sodeoka et al. have used the latter ligands in asymmetric fluorination of a wide range of substrates, including β-keto esters, β-keto phosphonates, oxindoles [38,40,43,51,53,56–57]. They have also recently reported the enantioselective fluorination of 3-(2-arylacetyl)-2-thiazolidinones with their extended catalytic system, NiCl_2_-BINAP/R_3_SiOTf-lutidine with high enantioselectivities [57]. This study is useful because, up until now, the fluorinated products obtained by Sodeoka's method have been prepared by diastereoselective methods [65–67]. Independently, our group has focused on the development of enantioselective fluorination and related reactions using bis(oxazoline) ligands, Box-Ph [(*S*,*S*)-2,2^'^-isopropylidene-bis(4-phenyl-2-oxazoline)] and DBFOX-Ph [(*R*,*R*)-4,6-dibenzofurandiyl-2,2'-bis(4-phenyloxazoline)] [33–34,36]. As an extension of this study, we herein evaluate our DBFOX-Ph/metal catalysis for the enantioselective fluorination of 3-(2-arylacetyl)-2-thiazolidinones with N-fluorobenzenesulfonimide (NFSI) (Figure 1).

## Results and Discussion

Our previous studies of the DBFOX-Ph/Ni(II)-catalyzed enantioselective fluorination of β-keto esters have shown that the optimal reaction conditions require NFSI as the fluorine source and a catalytic amount of Ni(ClO_4_)_2_·6H_2_O in CH_2_Cl_2_ at room temperature. Therefore, we first attempted the reaction of **1a** with the same conditions and found that the desired fluorinated product **2a** was obtained in 42% yield with 69% ee (Table 1, entry 1). The reaction at higher temperature (40 °C) improved the yield to 62% with slightly lower enantioselectivity (63% ee, entry 2). The reaction time in these experiments was shortened by the addition of 1 equiv of 2,6-lutidine and **2a** was obtained in 87% yield with 66% ee at room temperature (entry 3). Both yield and selectivity were improved to 90% and 74% ee when the reaction was performed at 0 °C (entry 4). The highest ee value of **2a** was obtained at −20 °C, but resulted in a decrease in yield (24%, 79% ee, entry 5). Changing the metal salts did not improve the results (entries 6 and 7). The absolute stereochemistry of **2a** was determined by comparing the optical rotation and HPLC analysis with the literature values [57]. Although the enantioselectivities are moderate to good in these examples (63–79% ee), the results are quite impressive because the fluorination proceeds even in the absence of base (entries 1 and 2). That is, both Ni(ClO_4_)_2_-DBFOX-Ph (unary system, entries 1 and 2) and Ni(ClO_4_)_2_-DBFOX-Ph/lutidine (binary system, entries 3–6) are moderately effective in the enantioselective fluorination of **1a**. According to the report by Sodeoka using their NiCl_2_-BINAP/R_3_SiOTf-lutidine (trinary system, up to 88% ee obtained), the reaction requires both R_3_SiOTf and lutidine [57]. They mentioned in the paper that a binary system consisting of Ni(OTf)_2_–binap complex and 2,6-lutidine failed to promote asymmetric fluorination. We also briefly attempted the fluorination of **1a** using the (*S*,*S*)-Box-Ph ligand instead of DBFOX-Ph. While the Box-Ph/Cu(OTf)_2_ catalyst was not effective (run 8), the Box-Ph/Ni(ClO_4_)_2_·6H_2_O catalyst gave the desired product **2a** in 33% yield with low enantioselectivity (15% ee, entry 9).

**Table 1:** Optimisation of the Conditions for DBFOX-Ph/Ni(II)-Catalysed Enantioselective Fluorination of 3-(2-Phenylacetyl)-2-thiazolidinone (**1a**)^a^.

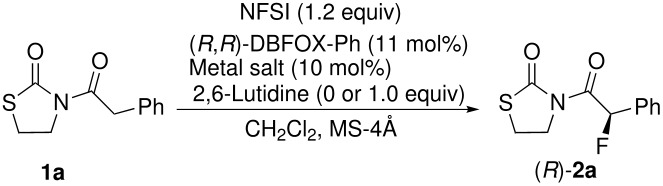						
Run	Metal salt	2,6- Lutidine (equiv)	Temp (°C)	Time	Yield (%)	ee (%)

1	Ni(ClO_4_)_2_·6H_2_O	none	rt	6 d	42	69
2	Ni(ClO_4_)_2_·6H_2_O	none	40	4 d	62	63
3	Ni(ClO_4_)_2_·6H_2_O	1.0	rt	17 h	87	66
4	Ni(ClO_4_)_2_·6H_2_O	1.0	0	20 h	90	74
5	Ni(ClO_4_)_2_·6H_2_O	1.0	−20	4 d	24	79
6	Ni(OAc)_2_·4H_2_O	1.0	rt	4 d	55	72
7	Zn(OAc)_2_	1.0	rt	3 d	NR	-
8^b,c^	Cu(OTf)_2_	1.0	0	2 d	NR	-
9^b^	Ni(ClO_4_)_2_·6H_2_O	1.0	0	2 d	33	15^d^

**^a^**For detailed reaction conditions, see Supporting Information File 1. Enantioselectivity was determined by chiral HPLC analysis. The absolute configuration of **2a** was determined by comparison with the optical rotation and HPLC analysis in the literature [57]. NR: No reaction. ^b^(*S*,*S*)-Box-Ph (11 mol%) was used instead of (*R*,*R*)-DBFOX-Ph. ^c^Ether was used as solvent. ^d^(*S*)-**2a** was obtained.

The DBFOX-Ph/Ni(ClO_4_)_2_·6H_2_O catalysis for fluorination showed high generality for various 3-(2-arylacetyl)-2-thiazolidinones **1a**–**k** in good to high yields with moderate to good enantioselectivities. The results are summarized in Table 2. The fluorination reaction was not very sensitive to substitution in the position of the phenyl group and the desired products with methoxy or methyl groups at the *o*-, *m*-, or *p*-position of the benzene ring were obtained in 65–78% ee (entries 2–7). The reactions of fluoro or bromo-substituted **1h**, **i** and bulky-substituted **1j**, **k** afforded the desired products **2h**–**k** in good yields with slightly lower enantioselectivities (56–62% ee, entries 8–11).

**Table 2:** Enantioselective Fluorination Reaction of 3-(2-Arylacetyl)-2-thiazolidinones with NFSI Catalyzed by DBFOX-Ph/Ni(II)^a^.

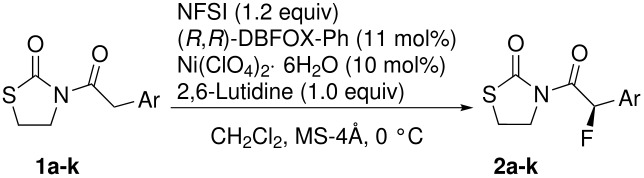						
Entry	**1**	Ar	**2**	Time (h)	Yield (%)	ee (%)

1	**1a**	Ph	**2a**	20	90	74
2	**1b**	C_6_H_4_-*o*-OMe	**2b**	48	96	78
3	**1c**	C_6_H_4_-*m*-OMe	**2c**	24	94	66
4	**1d**	C_6_H_4_-*p*-OMe	**2d**	24	90	65
5	**1e**	C_6_H_4_-*o*-Me	**2e**	48	69	76
6	**1f**	C_6_H_4_-*m*-Me	**2f**	48	75	73
7	**1g**	C_6_H_4_-*p*-Me	**2g**	48	75	77
8	**1h**	C_6_H_4_-*p*-F	**2h**	48	60	62
9	**1i**	C_6_H_4_-*p*-Br	**2i**	48	77	56
10	**1j**	1-Naphthyl	**2j**	48	85	59
11	**1k**	2-Naphthyl	**2k**	48	90	60

^a^For detailed reaction conditions, see Supporting Information File 1. Enantioselectivity was determined by chiral HPLC analysis. The absolute configuration of **2a** was determined by comparison with the optical rotation and HPLC analysis in the literature [57]. Others were tentatively assigned by comparing the signs of their optical rotations to that of **2a**.

**Scheme 1:**
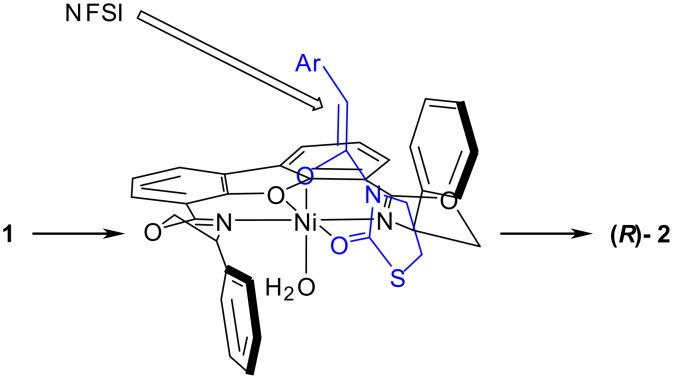
Transition-State Structure for the DBFOX-Ph/Ni(II) Catalyzed Enantioselective Fluorination of **1** to **2**.

The *R*-enantioselection of **2** can be explained by assuming an octahedral complex coordinated with a water molecule for DBFOX-Ph/Ni(II)/**1** as shown in Scheme 1. In the complex, the *Si* face is shielded by one of the phenyl groups of DBFOX-Ph so that NFSI approaches from the *Re* face of the substrates (Scheme 1). Since a major difference in ee values of **2** was not observed for the fluorination reaction of **1** with NFSI in the presence or absence of 2,6-lutidine (entries 1–3, Table 1), 2,6-lutidine presumably just accelerates the tautomerization of **1** to its enol form.

## Conclusion

This research has demonstrated that DBFOX-Ph/Ni(II) catalysis can be used for the catalytic enantioselective fluorination of 3-(2-arylacetyl)-2-thiazolidinones with or without 2,6-lutidine to afford chiral 2-fluoro-2-arylacetate derivatives in good to high yields with moderate to good enantioselectivities of up to 78% ee. The Box-Ph ligand was not effective for this reaction. Our best ee value is slightly lower than that of Sodeoka's report [57]; this is presumably due to the low activity of our catalyst system which requires higher reaction temperature conditions (0 °C vs. −20 °C [57]). Racemization of the products **2** during the fluorination reaction was ruled out since no racemization was observed when **2a** was stirred overnight under the same fluorination conditions. Further studies to improve the enantioselectivity of DBFOX-Ph/metal catalysis in enantioselective fluorination are under way.

## Supporting Information

File 1 Experimental methods. General methods, general procedure for the enantioselective catalytic fluorination, spectral data of **2**, copies of ^1^H, ^13^C and ^19^F-NMRs and HPLC charts of **2**
