# Supplementary material for: DBFOX-Ph/metal complexes: Evaluation as catalysts for enantioselective fluorination of 3-(2-arylacetyl)-2-thiazolidinones
Source: Beilstein J Org Chem. 2008 May 20;4:16. doi: 10.3762/bjoc.4.16 (PMC2486487; doi:10.3762/bjoc.4.16)
Supplement: File 1 — Experimental methods. General methods, general procedure for the enantioselective catalytic fluorination, spectral data of 2, copies of 1H, 13C and 19F-NMRs and HPLC charts of 2 [file Beilstein_J_Org_Chem-04-16-s001.doc]

**Supporting Information**

**Experimental methods**

**DBFOX-Ph/metal complexes: Evaluation as catalysts for enantioselective fluorination of 3-(2-arylacetyl)-2-thiazolidinones**

Takehisa Ishimaru, Norio Shibata*, Dhande Sudhakar Reddy,

Takao Horikawa, Shuichi Nakamura, Takeshi Toru*

Department of Frontier Materials, Graduate School of Engineering, Nagoya Institute of Technology, Gokiso, Showa-ku, Nagoya 466-8555, Japan.

**Experimental Section**

**General Methods**

All reactions were performed in oven-dried glassware under a positive pressure of nitrogen. Solvents were transferred *via* syringe and were introduced into the reaction vessels though a rubber septum. All of the reactions were monitored by thin-layer chromatography (TLC) carried out on 0.25 mm Merck silica-gel (60-F254). The TLC plates were visualized with UV light and 7% phosphomolybdic acid or *p*-anisaldehyde in ethanol/ heat. Column chromatography was carried out on a column packed with silica-gel 60N spherical neutral size 63–210 μm. The ^1^H-NMR (600 MHz), ^19^F-NMR (188 MHz), and ^13^C-NMR (150.9 MHz) spectra for solution in CDCl_3_ were recorded on a Buruker-600, a Varian Gemini-200. Chemical shifts (δ) are expressed in ppm downfield from internal TMS or CHCl_3_. HPLC analyses were performed on a JASCO PU-2080 Plus or SHIMADZU LC-2010A HT using 4.6 x 250 mm CHIRALPAK AD-H or CHIRALCEL OJ-H column. Mass spectra were recorded on a SHIMADZU GCMS-QP5050A. Optical rotations were measured on a HORIBA SEPA-300. Infrared spectra were recorded on a JASCO FT/ IR-200 spectrometer.

**General procedure for the Enantioselective Catalytic Fluorination**

Ni(ClO_4_)_2_⋅6H_2_O (10 mol%) and the (*R*,*R*)-4,6-dibenzofurandiyl-2,2’-bis(4-phenyloxazoline) (DBFOX-Ph) (11 mol%) were stirred under vacuum for 2 h at room temperature. Dry dichloromethane (2.0 ml) and MS 4Å (substrate/ MS 4A=1: 500 mol/ g) were added under nitrogen atmosphere and stirred for 1 h. Then **1** (0.1 mmol) was added directly to the catalyst solution. After stirring for another 20 min at 0 °C, NFSI (1.2 equiv) and 2,6-lutidine (1.0 equiv) were added to the mixture. The reaction was stirred at 0 °C until completion as indicated by TLC (24 h to 48 h), it was then stopped by addition of water. The reaction mixture was then diluted with dichloromethane, and the organic layer was washed with brine, dried over Na_2_SO_4_ and the solvent was evaporated under reduced pressure. The residue was purified by column chromatography on silica gel eluting with hexane/AcOEt/CHCl_3_ to give **2**. The ee of the product **2** was determined by chiral HPLC on CHIRALPAK AD-H or CHIRALCEL OJ-H column.

**3-[2-Fluoro-2-(phenyl)acetyl]-2-thiazolidinone (2a)**

White solid

^1^H-NMR (CDCl_3_, 600 MHz): 3.26–3.33 (m, 2H), 4.13–4.17 (m, 1H), 4.21–4.25 (m, 1H), 6.81 (d, *J*=48.6 Hz, 1H), 7.40–7.41 (m, 3H), 7.51–7.53 (m, 2H); ^13^C-NMR (CDCl_3_, 150.9 MHz): 172.5, 168.1 (d, *J*=26.7 Hz), 133.6 (d, *J*=20.1 Hz), 129.9 (d, *J*=3.0 Hz), 128.7 (d, *J*=1.5 Hz), 128.5 (d, *J*=4.6 Hz), 89.6 (d, *J*=179.3 Hz), 46.6, 25.6; ^19^F-NMR (CDCl_3_, 188 MHz): –170.3 (d, *J*=48.7 Hz, 1F); MS (EI): m/z 239 (M^+^); HPLC: (AD-H, hexane/ *^i^*PrOH=90/ 10, 1.0 ml/ min, 254 nm) t_R_ ((*R*)-isomer)=15.1 min, t_R_ ((*S*)-isomer)=17.5 min (74% ee); [α]_D_^25^ –69.2 (*c*=0.500, CHCl_3_ 74% ee), Lit. [1] [α]_D_^31^ –92.1 (*c*=1.0, CHCl_3_ 82% ee, *R*); IR (KBr): 2924, 2853, 1695, 1496, 1472, 1458, 1360, 1291, 1247, 1182, 1069, 1012, 967, 928, 858, 790, 768, 701, 654, 616 cm^–1^; Mp: 125–126 °C (CH_2_Cl_2_/ hexane)

**3-[2-Fluoro-2-(2-methoxyphenyl)acetyl]-2-thiazolidinone (2b)**

White solid

^1^H-NMR (CDCl_3_, 600 MHz): 3.27–3.34 (m, 2H), 3.89 (s, 3H), 4.18–4.27 (m, 2H), 6.98 (d, *J*=48.6 Hz, 1H), 6.94–6.97 (m, 2H), 7.24 (dt, *J*=7.8, 1.2 Hz, 1H), 7.37 (tt, *J*=8.1, 1.2 Hz, 1H); ^13^C-NMR (CDCl_3_, 150.9 MHz): 171.8, 168.4 (d, *J*=25.8 Hz), 157.9 (d, *J*=13.2 Hz), 131.4 (d, *J*=3.6 Hz), 129.0 (d, *J*=4.4 Hz), 122.4 (d, *J*=18.7 Hz), 120.6 (d, *J*=2.6 Hz), 111.4 (d, *J=*2.1 Hz), 85.9 (d, *J*=178.1 Hz), 55.9, 46.6, 25.7; ^19^F-NMR (CDCl_3_, 188 MHz): –173.7 (d, *J*=49.8 Hz, 1F); MS (EI): m/z 269 (M^+^); HPLC: (AD-H, hexane/ *^i^*PrOH=90/ 10, 1.0 ml/ min, 254 nm) t_R_ (minor-isomer)=21.5 min, t_R_ (major-isomer)=26.0 min (78% ee); [α]_D_^25^ –208.1 (*c*=0.500, CHCl_3_ 78% ee); IR (KBr): 3017, 2984, 2951, 2898, 2845, 1700, 1601, 1587, 1496, 1467, 1441, 1367, 1330, 1294, 1254, 1189, 1165, 1110, 1075, 1050, 1024, 1008, 967, 931, 865, 810, 769, 749, 685, 656, 607 cm^–1^; Mp: 119–120 °C (CH_2_Cl_2_/ hexane)

**3-[2-Fluoro-2-(3-methoxyphenyl)acetyl]-2-thiazolidinone (2c)**

White solid

^1^H-NMR (CDCl_3_, 600 MHz): 3.27–3.32 (m, 2H), 3.82 (d, *J*=4.2 Hz, 3H), 4.13–4.17 (m, 1H), 4.22 (dt, *J*=12.0, 7.8 Hz, 1H), 6.80 (d, *J*=48.6 Hz, 1H), 6.94 (d, *J*=7.2 Hz, 1H), 7.06 (t. *J*=1.2 Hz, 1H), 7.10 (d, *J*=7.2 Hz, 1H), 7.30 (t, *J*=7.8 Hz, 1H); ^13^C-NMR (CDCl_3_, 150.9 MHz): 172.5, 168.0 (d, *J*=26.6 Hz), 159.7, 134.9 (d, *J*=20.2 Hz), 129.8, 120.7 (d, *J*=5.0 Hz), 115.8 (d, *J*=2.9 Hz), 113.5 (d, *J*=4.8 Hz), 89.3 (d, *J*=179.7 Hz), 55.3, 46.7, 25.5; ^19^F-NMR (CDCl_3_, 188 MHz): –171.8 (d, *J*=47.2 Hz, 1F); MS (EI): m/z 269 (M^+^); HPLC: (AD-H, hexane/ *^i^*PrOH=90/ 10, 1.0 ml/ min, 254 nm) t_R_ (minor-isomer)=20.9 min, t_R_ (major-isomer)=23.0 min (66% ee); [α]_D_^25^ –54.8 (*c*=0.500, CHCl_3_ 66% ee); IR (KBr): 3019, 2969, 2938, 2883, 2716, 1688, 1600, 1490, 1472, 1454, 1434, 1362, 1326, 1288, 1250, 1204, 1169, 1079, 1035, 1013, 957, 890, 858, 825, 800, 759, 701, 687, 665, 653 cm^–1^; Mp: 97–98 °C (CH_2_Cl_2_/ hexane)

**3-[2-Fluoro-2-(4-methoxyphenyl)acetyl]-2-thiazolidinone (2d)**

White solid

^1^H-NMR (CDCl_3_, 600 MHz): 3.25–3.32 (m, 2H), 4.13–4.17 (m, 1H), 4.23 (dt, *J*=12.0, 7.8 Hz, 1H), 6.73 (d, *J*=48.6 Hz, 1H), 6.91 (d, *J*=7.8 Hz, 2H), 7.44 (dd, *J*=1.2, 8.4 Hz, 1H); ^13^C-NMR (CDCl_3_, 150.9 MHz): 172.3, 168.3 (d, *J*=27.5 Hz), 160.8 (d, *J*=2.9 Hz), 130.2 (d, *J*=4.1 Hz), 125.6 (d, *J*=21.0 Hz), 114.1 (d, *J*=1.8 Hz), 89.3 (d, *J*=179.0 Hz), 55.3, 46.6, 25.6; ^19^F-NMR (CDCl_3_, 188 MHz): –167.3 (d, *J*=48.7 Hz, 1F); MS (EI): m/z 269 (M^+^); HPLC: (AD-H, hexane/ *^i^*PrOH=90/ 10, 1.0 ml/ min, 254 nm) t_R_ (minor-isomer)=23.2 min, t_R_ (major-isomer)=28.1 min (65% ee); [α]_D_^25^ –84.5 (*c*=0.500, CHCl_3_ 65% ee); IR (KBr): 3052, 1970, 2932, 1720, 1686, 1610, 1586, 1512, 1456, 1370, 1326, 1309, 1280, 1242, 1200, 1178, 1116, 1075, 1034, 1005, 966, 926, 838, 825, 800, 769, 660, 626 cm^–1^; Mp: 142–143 °C (CH_2_Cl_2_/ hexane)

**3-[2-Fluoro-2-(2-methylphenyl)acetyl]-2-thiazolidinone (2e)**

White solid

^1^H-NMR (CDCl_3_, 600 MHz): 2.53 (d, *J*=1.8 Hz, 3H), 3.32 (t, *J*=7.8 Hz, 2H), 4.24–4.30 (m, 2H), 6.90 (d, *J*=49.8 Hz, 1H), 7.16–7.20 (m, 2H), 7.26 (d, *J*=7.2 Hz, 1H), 7.31 (tt, *J*=2.4, 7.2 Hz, 1H); ^13^C-NMR (CDCl_3_, 150.9 MHz): 172.2, 168.4 (d, *J*=25.5 Hz), 138.9 (d, *J*=3.3 Hz), 131.8 (d, *J*=17.7 Hz), 131.1 (d, *J*=2.7 Hz), 130.1 (d, *J*=4.1 Hz), 127.4 (d, *J*=3.6 Hz), 126.3 (d, *J*=2.9 Hz), 87.9 (d, *J*=178.8 Hz), 46.5, 25.7, 18.9; ^19^F-NMR (CDCl_3_, 188 MHz): –170.9 (d, *J*=48.5 Hz, 1F); MS (EI): m/z 253 (M^+^); HPLC: (AD-H, hexane/ *^i^*PrOH=90/ 10, 1.0 ml/ min, 254 nm) t_R_ (minor-isomer)=11.3 min, t_R_ (major-isomer)=14.0 min (76% ee); [α]_D_^25^ –193.6 (*c*=0.500, CHCl_3_ 76% ee); IR (KBr): 2970, 2932, 2855, 1699, 1495, 1471, 1450, 1361, 1316, 1291, 1248, 1174, 1068, 1006, 965, 929, 863, 813, 762, 730, 654, 605 cm^–1^; Mp: 114–115 °C (CH_2_Cl_2_/ hexane)

**3-[2-Fluoro-2-(3-methylphenyl)acetyl]-2-thiazolidinone (2f)**

White solid

^1^H-NMR (CDCl_3_, 600 MHz): 2.37 (s, 3H), 3.26–3.32 (m, 2H), 4.13–4.18 (m, 1H), 4.23 (dt, *J*=12.6, 7.8 Hz, 1H), 6.77 (d, *J*=49.2 Hz, 1H), 7.21 (dd, *J*=0.6, 7.2 Hz, 1H), 7.28 (dd, *J*=7.2, 7.8 Hz, 1H), 7.30–7.33 (m, 2H); ^13^C-NMR (CDCl_3_, 150.9 MHz): 172.4, 168.2 (d, *J*=26.7 Hz), 138.6 (d, *J*=1.7 Hz), 133.4 (d, *J*=19.9 Hz), 130.7 (d, *J*=3.2 Hz), 129.0 (d, *J*=4.5 Hz), 128.6 (d, *J*=1.5 Hz), 125.6 (d, *J*=4.7 Hz), 89.6 (d, *J*=179.0 Hz), 46.6, 25.5, 21.3; ^19^F-NMR (CDCl_3_, 188 MHz): –169.9 (d, *J*=48.7 Hz, 1F); MS (EI): m/z 253 (M^+^); HPLC: (OJ-H, hexane/ *^i^*PrOH=90/ 10, 1.0 ml/ min, 254 nm) t_R_ (major-isomer)=32.7 min, t_R_ (minor-isomer)=39.2 min (73% ee); [α]_D_^25^ –77.5 (*c*=0.500, CHCl_3_ 73 % ee); IR (KBr): 3003, 2970, 2925, 2855, 1699, 1495, 1471, 1450, 1361, 1291, 1248, 1174, 1068, 1006, 929, 863, 813, 762, 730, 654, 605 cm^–1^; Mp: 93–94 °C (CH_2_Cl_2_/ hexane)

**3-[2-Fluoro-2-(4-methylphenyl)acetyl]-2-thiazolidinone (2g)**

White solid

^1^H-NMR (CDCl_3_, 600 MHz): 2.36 (d, *J*=1.8 Hz, 3H), 3.25–3.32 (m, 2H), 4.12–4.18 (m, 1H), 4.22 (dt, *J*=12.0, 7.8 Hz, 1H), 6.76 (d, *J*=48.6 Hz, 1H), 7.20 (d, *J*=7.8 Hz, 2H), 7.40 (dd, *J*=1.2, 7.8 Hz, 1H); ^13^C-NMR (CDCl_3_, 150.9 MHz): 172.4, 168.3 (d, *J*=31.1 Hz), 140.1 (d, *J*=3.2 Hz), 130.6 (d, *J*=20.4 Hz), 129.4 (d, *J*=1.7 Hz), 128.5 (d, *J*=4.5 Hz), 89.5 (d, *J*=89.5 Hz), 46.6, 25.6, 21.3; ^19^F-NMR (CDCl_3_, 188 MHz): –169.1 (d, *J*=48.7 Hz, 1F); MS (EI): m/z 253 (M^+^); HPLC: (AD-H, hexane/ *^i^*PrOH=90/ 10, 1.0 ml/ min, 254 nm) t_R_ (major-isomer)=12.3 min, t_R_ (minor-isomer)=13.9 min (77% ee); [α]_D_^25^ –72.9 (*c*=0.500, CHCl_3_ 77 % ee); IR (KBr): 2994, 2961, 2922, 1717, 1698, 1610, 1513, 1472, 1446, 1364, 1287, 1266, 1241, 1183, 1066, 1008, 926, 860, 847, 827, 797, 766, 723, 661, 653 cm^–1^; Mp: 92–93 °C (CH_2_Cl_2_/ hexane)

**3-[2-Fluoro-2-(4-fluorophenyl)acetyl]-2-thiazolidinone (2h)**

White solid

^1^H-NMR (CDCl_3_, 600 MHz): 3.28–3.35 (m, 2H), 4.15 (ddd, *J*=5.4, 7.8, 12.0 Hz, 1H), 4.24 (td, *J*=12.0, 7.8 Hz, 1H), 6.77 (d, *J*=48.0 Hz, 1H), 7.08 (dt, *J*=0.6, 8.4 Hz, 2H), 7.51–7.53 (m, 2H); ^13^C-NMR (CDCl_3_, 150.9 MHz): 172.6, 168.0 (d, *J*=26.9 Hz), 163.6 (dd, *J*=3.2, 249.7 Hz), 130.6 (dd, *J*=4.5, 8.6 Hz), 129.5 (dd, *J*=3.3, 20.8 Hz), 115.8 (dd, *J*=1.7, 21.7 Hz), 88.7 (d, *J*=179.7 Hz), 46.6, 25.6; ^19^F-NMR (CDCl_3_, 188 MHz): –169.2 (dd, *J*=5.3, 47.2 Hz 1F), –110.2 – –110.1 (m, 1F); MS (EI): m/z 257 (M^+^); HPLC: (AD-H, hexane/ *^i^*PrOH=90/ 10, 1.0 ml/ min, 254 nm) t_R_ (major-isomer)=12.0 min, t_R_ (minor-isomer)=17.4 min (62% ee); [α]_D_^25^ –51.3 (*c*=0.500, CHCl_3_ 62 % ee); IR (neat): 2950, 1694, 1591, 1489, 1445, 1408, 1360, 1286, 1242, 1178, 1072, 1011, 968, 922, 859, 827, 782, 709, 657, 616 cm^–1^; Mp: 84–85 °C (CH_2_Cl_2_/ hexane)

**3-[2- Fluoro-2- (4-Bromophenyl)acetyl]-2-thiazolidinone (2i)**

Pale yellow oil

^1^H-NMR (CDCl_3_, 600 MHz): 3.29–3.35 (m, 2H), 4.12–4.18 (m, 1H), 4.24 (dt, *J*=12.0, 8.4 Hz, 1H), 6.76 (d, *J*=48.6, 1H), 7.41 (dd, *J*=1.8, 8.4 Hz, 2H), 7.54 (d, *J*=8.4 Hz, 2H); ^13^C-NMR (CDCl_3_, 150.9 MHz): 172.7, 167.7 (d, *J*=26.6 Hz), 132.5 q(d, *J*=20.7 Hz), 132.0, 130.1 (d, *J*=4.6 Hz), 124.4 (d, *J*=3.6 Hz), 88.8 (d, *J*=180.0 Hz), 46.6, 25.6; ^19^F-NMR (CDCl_3_, 188 MHz): –171.1 (d, *J*=47.4 Hz, 1F); MS (EI): m/z 317, 319 (M^+^); HPLC: (AD-H, hexane/ *^i^*PrOH=90/ 10, 1.0 ml/ min, 254 nm) t_R_ (major-isomer)=13.4 min, t_R_ (minor-isomer)=20.4 min (56% ee); [α]_D_^25^ –33.8 (*c*=0.400, CHCl_3_ 56 % ee); IR (neat): 3074, 2924, 2854, 1720, 1690, 1603, 1508, 1447, 1421, 1368, 1285, 1228, 1186, 1161, 1102, 1077, 1019, 928, 893, 867, 845, 810, 779, 725, 658 cm^–1^

**3-[2-Fluoro-2-(1-naphthyl)acetyl]-2-thiazolidinone (2j)**

White solid

^1^H-NMR (CDCl_3_, 600 MHz): 3.21–3.29 (m, 2H), 4.19–4.23 (m, 1H), 4.26 (dt, *J*=12.0, 8.4 Hz, 1H), 7.44 (d, *J*=49.2 Hz, 1H), 7.44 (dt, *J*=1.2, 7.8 Hz, 1H), 7.48–7.50 (m, 1H), 7.54 (dt, *J*=0.6, 7.5 Hz, 1H), 7.60–7.63 (m, 1H), 7.88 (d, *J*=8.4 Hz, 1H), 7.91 (d, *J*=8.4 Hz, 1H), 8.30 (d, *J*=8.4 Hz, 1H); ^13^C-NMR (CDCl_3_, 150.9 MHz): 172.3, 168.5 (d, *J*=25.7 Hz), 133.9, 131.5, 131.0 (d, *J*=3.8 Hz), 129.5 (d, *J*=18.1 Hz), 128.7, 127.2, 127.0 (d, *J*=5.6 Hz), 126.3, 124.9 (d, *J*=2.7 Hz), 123.6, 88.1 (d, *J*=180.0 Hz), 46.7, 25.6; ^19^F-NMR (CDCl_3_, 188 MHz): –170.4 (d, *J*=48.7 Hz, 1F); MS (EI): m/z 289 (M^+^); HPLC: (AD-H, hexane/ *^i^*PrOH=90/ 10, 1.0 ml/ min, 254 nm) t_R_ (minor-isomer)=16.2 min, t_R_ (major-isomer)=21.2 min (59% ee); [α]_D_^25^ –184.0 (*c*=0.500, CHCl_3_ 59 % ee); IR (KBr): 3060, 2966, 2632, 1717, 1698, 1599, 1510, 1471, 1446, 1361, 1346, 1289, 1240, 1131, 1004, 927, 858, 821, 807, 781, 750, 678, 638, 616 cm^–1^; Mp: 158–160 °C

**3-[2-Fluoro-2-(2-naphthyl)acetyl]-2-thiazolidinone (2k)**

Yellow oil

^1^H-NMR (CDCl_3_, 600 MHz): 3.24–3.31 (m, 2H), 4.16 (ddd, *J*=5.4, 7.8, 12.0 Hz, 1H), 4.24 (td, *J*=7.8, 12.0 Hz, 1H), 6.98 (d, *J*=48.6 Hz, 1H), 7.50–7.55 (m, 2H), 7.62 (d, *J*=1.2, 8.4 Hz, 1H), 7.84–7.89 (m, 3H), 8.02 (s, 1H); ^13^C-NMR (CDCl_3_, 150.9 MHz): 172.5, 168.1 (d, *J*=26.7 Hz), 133.8, 132.9, 130.9 (d, *J*=19.9 Hz), 128.7 (d, *J*=5.9 Hz), 128.6, 128.5, 127.7, 127.1, 126.5, 125.1 (d, *J*=3.6 Hz), 89.7 (d, *J*=179.6 Hz), 46.7, 25.5; ^19^F-NMR (CDCl_3_, 188 MHz): –170.2 (d, *J*=48.7 Hz, 1F); MS (EI): m/z 289 (M^+^); HPLC: (AD-H, hexane/ *^i^*PrOH=98/ 2, 1.0 ml/ min, 254 nm) t_R_ (minor-isomer)=56.6 min, t_R_ (major-isomer)=63.3 min (60% ee); [α]_D_^25^ –90.2 (*c*=0.500, CHCl_3_ 60 % ee); IR (neat): 3057, 2939, 1696, 1509, 1470, 1444, 1360, 1286, 1236, 1182, 1071, 1019, 957, 924, 862, 818, 799, 761, 657 cm^–1^

### Reference

1. Suzuki, T.; Hamashima, Y.; Sodeoka, M. *Angew. Chem., Int. Ed.* **2007,** *46,* 5435–5439. doi:[10.1002/anie.200701071](http://dx.doi.org/10.1002/anie.200701071)

**HPLC charts**

HPLC using an AD-H column

**2a** (*n*-hexane/*i*-PrOH=90/10, flow rate 1.0 mL/ min, λ=254 nm)

| No | TIME (min) | AREA (%) | HEIGHT (%) |
| --- | --- | --- | --- |
| 1 | 15.1 | 86.991 | 88.617 |
| 2 | 17.5 | 13.009 | 11.387 |

HPLC using an AD-H column

**2b** (*n*-hexane/*i*-PrOH=90/10, flow rate 1.0 mL/ min, λ=254 nm)

**Racemic compound of 2b**

| No | TIME (min) | AREA (%) | HEIGHT (%) |  | No | TIME (min) | AREA (%) | HEIGHT (%) |
| --- | --- | --- | --- | --- | --- | --- | --- | --- |
| 1 | 22.3 | 49.247 | 53.818 |  | 1 | 21.5 | 10.979 | 12.714 |
| 2 | 27.4 | 50.753 | 46.182 |  | 2 | 26.0 | 89.021 | 87.286 |

HPLC using an AD-H column

**2c** (*n*-hexane/*i*-PrOH=90/10, flow rate 1.0 mL/ min, λ=254 nm)

**Racemic compound of 2c**

| No | TIME (min) | AREA (%) | HEIGHT (%) |  | No | TIME (min) | AREA (%) | HEIGHT (%) |
| --- | --- | --- | --- | --- | --- | --- | --- | --- |
| 1 | 18.5 | 50.259 | 52.284 |  | 1 | 20.9 | 83.004 | 83.732 |
| 2 | 19.9 | 49.741 | 47.716 |  | 2 | 23.0 | 16.996 | 16.268 |

HPLC using an AD-H column

**2d** (*n*-hexane/*i*-PrOH=90/10, flow rate 1.0 mL/ min, λ=254 nm)

**Racemic compound of 2d**

| No | TIME (min) | AREA (%) | HEIGHT (%) |  | No | TIME (min) | AREA (%) | HEIGHT (%) |
| --- | --- | --- | --- | --- | --- | --- | --- | --- |
| 1 | 24.7 | 55.667 | 60.523 |  | 1 | 23.2 | 82.267 | 84.968 |
| 2 | 30.1 | 44.333 | 39.477 |  | 2 | 28.1 | 17.733 | 15.032 |

HPLC using an AD-H column

**2e** (*n*-hexane/*i*-PrOH=90/10, flow rate 1.0 mL/ min, λ=254 nm)

**Racemic compound of 2e**

| No | TIME (min) | AREA (%) | HEIGHT (%) |  | No | TIME (min) | AREA (%) | HEIGHT (%) |
| --- | --- | --- | --- | --- | --- | --- | --- | --- |
| 1 | 11.6 | 51.631 | 55.073 |  | 1 | 11.3 | 12.003 | 14.329 |
| 2 | 14.4 | 48.369 | 44.927 |  | 2 | 14.0 | 87.997 | 85.671 |

HPLC using an OJ-H column

**2f** (*n*-hexane/*i*-PrOH=90/10, flow rate 1.0 mL/ min, λ=254 nm)

**Racemic compound of 2f**

| No | TIME (min) | AREA (%) | HEIGHT (%) |  | No | TIME (min) | AREA (%) | HEIGHT (%) |
| --- | --- | --- | --- | --- | --- | --- | --- | --- |
| 1 | 32.8 | 49.934 | 54.864 |  | 1 | 32.7 | 86.627 | 88.671 |
| 2 | 39.2 | 50.066 | 45.136 |  | 2 | 39.2 | 13.373 | 11.329 |

HPLC using an AD-H column

**2g** (*n*-hexane/*i*-PrOH=90/10, flow rate 1.0 mL/ min, λ=254 nm)

**Racemic compound of 2g**

| No | TIME (min) | AREA (%) | HEIGHT (%) |  | No | TIME (min) | AREA (%) | HEIGHT (%) |
| --- | --- | --- | --- | --- | --- | --- | --- | --- |
| 1 | 12.1 | 54.801 | 57.653 |  | 1 | 12.3 | 88.510 | 89.598 |
| 2 | 13.8 | 45.199 | 42.347 |  | 2 | 13.9 | 11.490 | 10.402 |

HPLC using an AD-H column

**2h** (*n*-hexane/*i*-PrOH=90/10, flow rate 1.0 mL/ min, λ=254 nm)

**Racemic compound of** 2h

| No | TIME (min) | AREA (%) | HEIGHT (%) |  | No | TIME (min) | AREA (%) | HEIGHT (%) |
| --- | --- | --- | --- | --- | --- | --- | --- | --- |
| 1 | 12.1 | 50.433 | 59.358 |  | 1 | 12.0 | 80.761 | 85.462 |
| 2 | 17.6 | 49.567 | 40.642 |  | 2 | 17.4 | 19.239 | 14.538 |

HPLC using an AD-H column

**2i** (*n*-hexane/*i*-PrOH=90/10, flow rate 1.0 mL/ min, λ=254 nm)

**Racemic compound of 2i**

| No | TIME (min) | AREA (%) | HEIGHT (%) |  | No | TIME (min) | AREA (%) | HEIGHT (%) |
| --- | --- | --- | --- | --- | --- | --- | --- | --- |
| 1 | 13.6 | 52.038 | 61.287 |  | 1 | 13.4 | 78.212 | 84.052 |
| 2 | 20.5 | 47.962 | 38.713 |  | 2 | 20.4 | 21.788 | 15.948 |

HPLC using an AD-H column

**2j** (*n*-hexane/*i*-PrOH = 90/10, flow rate 1.0 mL/min, λ = 254 nm)

**Racemic compound of 2j**

| No | TIME (min) | AREA (%) | HEIGHT (%) |  | No | TIME (min) | AREA (%) | HEIGHT (%) |
| --- | --- | --- | --- | --- | --- | --- | --- | --- |
| 1 | 16.3 | 50.617 | 56.054 |  | 1 | 16.2 | 20.496 | 25.926 |
| 1 | 21.2 | 49.383 | 43.946 |  | 1 | 21.2 | 79.504 | 74.074 |

HPLC using an AD-H column

**2k** (*n*-hexane/*i*-PrOH = 90/10, flow rate 1.0 mL/min, λ = 254 nm)

**Racemic compound of 2k**

| No | TIME (min) | AREA (%) | HEIGHT (%) |  | No | TIME (min) | AREA (%) | HEIGHT (%) |
| --- | --- | --- | --- | --- | --- | --- | --- | --- |
| 1 | 57.0 | 50.091 | 54.836 |  | 1 | 56.6 | 79.966 | 82.922 |
| 2 | 63.7 | 49.909 | 45.164 |  | 2 | 63.3 | 20.034 | 17.078 |

**NMR spectra**
